# Salivary cortisol as a marker for assessing the problem‐focused coping style of stressed students during the first year of university: An experimental study

**DOI:** 10.1002/hsr2.1280

**Published:** 2023-06-05

**Authors:** Mitsuo Nagane, Yoshinori Oyama, Fuminobu Tamalu, Naofumi Miwa

**Affiliations:** ^1^ Department of Physiology Saitama Medical University Moroyama‐machi Saitama Japan; ^2^ Department of Educational Psychology Chiba University Chiba‐shi Chiba Japan

**Keywords:** coping styles (problem‐focused, emotion‐focused, and escape‐focused), first‐year university students, hypothalamic‐pituitary‐adrenal (HPA) axis, salivary cortisol, stress paradigm, α‐amylase

## Abstract

**Background and Aims:**

First‐year students encounter substantial stress when they enter university. Their mental health often depends upon how well they cope with the stress of university life. Salivary components are well known to reflect the stress status of the students; however, the relationship between salivary components and coping styles remains unknown.

**Methods:**

In this study, 54 healthy first‐year students voluntarily completed a questionnaire that addressed three different coping styles: problem‐focused, emotion‐focused, and escape‐focused. We simultaneously collected salivary samples from students in the classroom and measured concentrations of salivary cortisol and α‐amylase by enzyme‐linked immunosorbent assays over 4 months.

**Results:**

We examined the relationship between coping style and salivary cortisol concentrations and found that the mean salivary cortisol concentrations were significantly lower in students who had a higher Likert‐type score for the problem‐focused coping style than in students who had a lower score. The difference in the mean cortisol concentrations between the two groups increased over time. However, we observed no apparent correlation between α‐amylase concentrations and Likert scores of the three coping styles.

**Conclusion:**

These results suggest that salivary cortisol concentrations might reflect the stress‐coping status, particularly involving the problem‐focused coping style.

## 1 BACKGROUND

First‐year university students experience substantial stress upon entrance to the university.^1^, ^2^, ^3^ The transition to university life forces them to adapt to multiple changes that involve both personal/affective and social/professional components. These daily stressors have been known to couple with poor physical and psychological health among university students.^4^ Recent reports have shown that the stress during this transition can impede learning and mature behavior, as well as the acquisition of social competence. Furthermore, chronic failure to cope with stress is a risk factor for some illnesses, such as psychophysiological disorders.^5^, ^6^ Therefore, knowing their coping status can help promote a more comfortable and productive time on campus.

Following the proposal of Lazarus and Folkman's theoretical foundation,^7^, ^8^ several stress coping styles have become well known, notably problem‐focused coping, emotion‐focused coping, and escape‐focused coping.^9^, ^10^, ^11^ The problem‐focused coping style aims to reduce the amount of stress by changing the source of the stress. The emotion‐focused coping style manages emotional responses to stressful events. Escape‐focused coping is a defensive reaction to a situation that is perceived as threatening and difficult.^11^

Psychophysiological studies have revealed that all strategies designed to cope with stress are related to the hypothalamic‐pituitary‐adrenal (HPA) axis^5^ and the autonomic nervous system (ANS) function, with cortisol and α‐amylase as representative biomarkers of the HPA axis and the ANS, respectively.

The concentration of salivary cortisol in adolescents increased in response to stronger‐than‐usual perceived stress when they were closely engaged with the source of daily stress.^12^ Meanwhile, salivary α‐amylase has emerged as a valid and reliable marker of ANS activity in response to stressors.^13^ However, the continuous monitoring of salivary cortisol and α‐amylase of first‐year students since entering university has not yet been examined, and major questions remain to be answered, such as (i) whether salivary cortisol or a‐amylase reflects the coping status of first‐year students, (ii) which of cortisol or a‐amylase more accurately reflect the students' stress coping styles, and (iii) which of the three coping styles (problem‐focused, emotion‐focused, escape‐focused) correlates with salivary components.

We previously found that changes in the concentrations of salivary components (e.g., cortisol and melatonin) were associated with psychosomatic conditions in university students and disrupted circadian rhythms.^14^, ^15^ However, we did not examine the relationships between salivary components and daily stress coping style. In the current study, we hypothesized that coping styles might differ among first‐year students; such styles might also correlate with salivary cortisol and α‐amylase concentrations. Thus, we used a questionnaire to evaluate the status of each of the three stress coping styles (i.e., problem‐focused, emotion‐focused, and escape‐focused); we also determined the concentrations of salivary cortisol and α‐amylase over a period of 4 months. Subsequently, we investigated relationships between the level of stress adaptation for each coping style and the concentrations of cortisol and α‐amylase when individuals adapted to new circumstances.

## 2 METHODS

### 2.1 Participants and study design

The first semester begins in April and ends at the end of July at Chiba University in Japan. The participants were 54 (16 men and 38 women) physically healthy Japanese university students from the Faculty of Education; all participants had the same course schedules. Their ages ranged from 18 to 20 years, and all participants provided written informed consent. The Ethics Committee of Chiba University approved the study protocol.

### 2.2 Likert‐type coping styles checklist

We used 14 items from Ozeki's validated Japanese version of the coping style questionnaire obtained by factorial analysis.^16^ Each self‐reported questionnaire was scored using a four‐point Likert‐type scale that measured each participant's preferred coping style: 1 = “not used,” 2 = “not frequently used,” 3 = “sometimes used,” and 4 = “regularly used.” Participants completed these reports once per month for 4 months. The survey was conducted in the first week of every month so that the intervals between surveys were almost the same. The 14 items were grouped into the three coping styles: problem‐focused (five items), emotion‐focused (three items), and escape‐focused (six items) (see Appendix). We regarded these three coping styles as mental health adaptations.

### 2.3 Salivary data analysis

The experiments were conducted at approximately the same time of day (around noon) at the end of the same class once per month (from April to July). All participants were non‐smokers and non‐alcohol drinkers. Saliva was collected by the participants in Salivette sampling tubes (Sarstedt) using polyester swabs after 2 min of chewing and stored at −20°C until use. These samples were assayed with a Cortisol Enzyme Immunoassay Kit and α‐Amylase Kinetic Enzyme Assay Kit (Salimetrics; LLC). Salivary cortisol concentrations were classified into two groups: low and high. The low group represented cortisol concentrations below mean − 0.5 SD, and the high group represented concentrations above mean + 0.5 SD.

### 2.4 Data analysis

**Figure 1:**
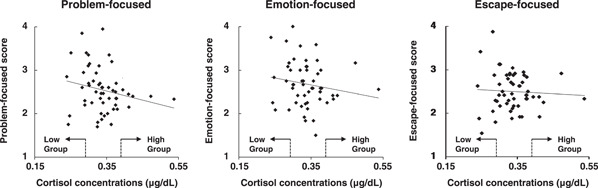
Relationship between coping style and cortisol concentration. Multiple correlation coefficients are shown. Left: problem‐focused, *R* ^2^ = 0.047, middle: emotion‐focused, *R* ^2^ = 0.027, right: escape‐focused, *R* ^2^ = 0.001. High group: >mean + 0.5 SD (0.34 + 0.05); low group: <mean − 0.5 SD (0.34−0.05).

**Figure 2:**
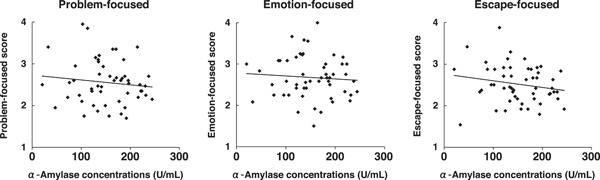
Relationship between coping style and α‐amylase concentration. Left: problem‐focused, *R* ^2^ = 0.014, middle: emotion‐focused, *R* ^2^ = 0.005, right; escape‐focused, *R* ^2^ = 0.035.

SPSS software (IBM Statistics; Version 21.0 for Mac; IBM Corp.) was used for all analyses. Correlation coefficients between the level of stress adaptation for each coping style and the concentrations of salivary cortisol and α‐amylase were assayed using scatter plots (Figures 1 and 2).

**Figure 3:**
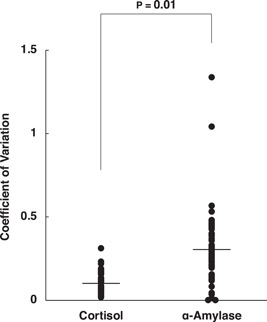
Coefficient of variation (CV) between cortisol and α‐amylase concentrations. The CV was calculated for Student's *t*‐test between the cortisol (*M* = 0.102, SD = 0.060) and α‐amylase concentrations (*M* = 0.305, SD = 0.229). *t* = −6.257, *p* = 0.01.

**Figure 4:**
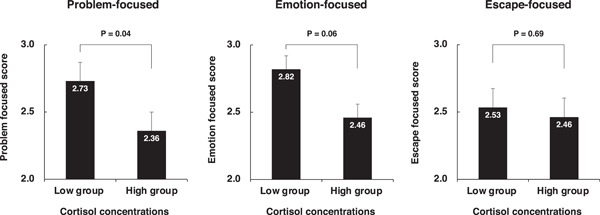
Comparison of coping style scores between the high and low cortisol groups. High or low groups: Participants (*n* = 54) were assigned to the high (*n* = 16) or low group (*n* = 18) based on their coping style points. Error bars represent standard errors. Student's *t*‐test, problem‐focused, *t* = 2.04, *p* = 0.04, emotion‐focused, *t* = 1.87, *p* = 0.06, escape‐focused, *t* = 0.39, *p* = 0.69.

**Figure 5:**
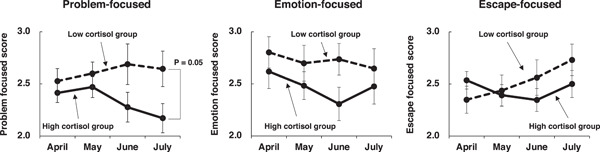
Time course of each coping style from April to July. Participants (*n* = 54) were assigned to the high (solid line) or low groups (dashed line) based on their cortisol concentration. Analysis of variance was performed between the two groups: problem‐focused, *F* = 6.85, *p* < 0.05; emotion‐focused, *F* = 4.23, *p* < 0.05; escape‐focused, *F* = 0.46, n.s., respectively.

The statistical significance between the cortisol and α‐amylase concentrations converted to the coefficient of variation; (CV; standard deviation [SD] divided by the mean) was determined using the Student *t*‐test (Figure 3). Repeated analysis of variance was performed on the participants' cortisol concentrations to compare the high and low group in each of the coping styles. Post hoc *t*‐tests at each time point were performed to assess group differences (Figures 4 and 5). A two‐tailed *p*‐value < 0.05 was considered statistically significant.

## 3 RESULTS

We first compared the concentrations of salivary cortisol and α‐amylase with the level of stress adaptation for each coping style (i.e., problem‐focused, emotional‐focused, and escape‐focused). Our scatter plot analyses revealed that the concentration of salivary cortisol was weakly correlated with the level of the problem‐focused coping style (*R* ^2^ = 0.047, *n* = 54) (Figure 1, left), but it was not correlated with the emotion‐focused coping style (*R* ^2^ = 0.027, *n* = 54) or the escape‐focused coping style (*R* ^2^ = 0.001, *n* = 54). Scatter plot analyses showed no correlations between α‐amylase concentrations and any of the three coping styles (problem‐focused, *R* ^2^ = 0.014, *n* = 53; emotion‐focused, *R* ^2^ = 0.005, *n* = 53; and escape‐focused, *R* ^2^ = 0.035, *n* = 53) (Figure 2).

To characterize the fluctuation of both cortisol and α‐amylase concentrations, we examined the CV between cortisol and α‐amylase, as shown in Figure 3. Our results revealed a significant difference (*p* = 0.01) in the CV between cortisol and α‐amylase. The data variability was greater for α‐amylase than for cortisol.

A weak correlation was detected between salivary cortisol concentrations and the problem‐focused coping style (Figure 1, left). Thus, we investigated the relationship between the two by generating two groups according to the mean Likert‐type score for the problem‐focused coping style (higher >mean + 0.5 SD; lower <mean − 0.5 SD); we calculated the mean salivary cortisol concentrations in each group for comparison. The results showed that students with a higher Likert‐type score exhibited lower concentrations (2.36) of salivary cortisol compared with students who had a lower Likert‐type score (2.73) (*p* = 0.04, *n* = 54, unpaired Student *t*‐test, Figure 5, left). Similar analyses were conducted for the other two coping styles. The emotion‐focused coping style was weakly correlated with salivary cortisol concentrations (*p* = 0.06, *n* = 54, Figure 5, middle). The escape‐focused coping style showed no apparent correlation with salivary cortisol concentrations (*p* = 0.69, *n* = 54, Figure 5, right).

To further characterize the relationship between problem‐focused coping and salivary cortisol concentrations, we monitored the relationship over 4 months (April to July). In April, no apparent difference in mean cortisol concentrations was present between the higher and lower Likert‐type score groups; however, an increasing difference was observed over time, such that it was statistically significant in July (2.17 for the higher group, 2.64 for the lower, *p* = 0.05, unpaired Student *t*‐test). Similar time course analyses for the emotion‐focused and escape‐focused coping styles revealed no apparent correlations with salivary cortisol concentrations (Figure 5, middle and right).

We performed similar analyses for α‐amylase concentrations by generating two (i.e., high and low) groups according to the Likert‐type scores; however, we observed no correlation with the Likert‐type score for any of the coping styles (data not shown). Thus, salivary cortisol concentrations reflected the degree of problem‐focused coping; it could potentially be correlated with the degree of emotion‐focused coping but not escape‐focused coping. Salivary α‐amylase was not correlated with any of the coping styles.

## 4 DISCUSSION

### 4.1 Cortisol and α‐amylase as biomarkers

Our results indicate that first‐year university students who adopted a problem‐focused coping style tended to have lower concentrations of salivary cortisol, suggesting that downregulation of the HPA axis is correlated with better problem‐focused coping.

This suggestion is consistent with the results of previous studies. For example, Matheson and Cole^17^ reported that problem‐focused coping was associated with a sense of optimism, along with a perception of the consequences of a devalued identity.

Furthermore, Sladek et al.^18^ reported that responding to stress by using social support was linked to lower cortisol responses to social stress.

The problem‐focused coping style has also been associated with reduced neuroendocrine reactivity (including the HPA axis) among younger people. Accordingly, we expected that salivary cortisol concentration would be correlated with psychological status when using the problem‐focused coping style; it could serve as a biomarker for the problem‐focused coping style.

As stated, no significant differences in α‐amylase concentrations were observed in any of the coping styles (Figure 2). Notably, the SD of α‐amylase concentrations was significantly greater than the SD of cortisol concentrations (Figure 3), suggesting greater individual variation in responses, possibly because of inherent physiological characteristics. Salivary α‐amylase concentrations tended to vary substantially; thus, α‐amylase may not be a useful biomarker to monitor the psychophysiological response of a student group.

### 4.2 Different correlations between cortisol and each of the three coping styles are not strictly separated

We found that salivary cortisol concentrations were significantly correlated with the high and low scores of the problem‐focused coping style but only weakly correlated with the high and low scores of the emotion‐focused coping style. No correlations were detected with the escape‐focused coping style (*p* = 0.06, *p* = 0.04, and *p* = 0.06 for problem‐focused, emotion‐focused, and escape‐focused coping scores, respectively, Figure 4). Notably, the extent of correlations was similar between the problem‐focused and emotion‐focused coping styles. Emotion‐focused coping is sometimes related to better adjustment to a stressor, similar to the problem‐focused coping style.^19^ Therefore, these two coping styles comprise a group of similar adaptive behaviors to a stressor; they may share some control centers in the central nervous system which modify the HPA axis.

The escape‐focused strategy is generally presumed to represent a poor adjustment to a stressor.^19^, ^20^, ^21^ Figure 5 contrasts the escape‐focused style with the other two styles. The characteristics of the stress paradigm in the escape‐focused coping style differ from those of the stress paradigm in the other two styles: a defensive reaction to stress in the escape‐focused coping style versus management of stress in the other two styles. This difference toward the stressor may reflect a difference in the neuroendocrine control center that modifies individual behavior during the stress paradigm. We suspect that escape‐focused coping may not be accompanied by an HPA‐axis response, which could explain the different correlations with salivary cortisol concentrations.

### 4.3 Limitations and future studies

First of all, further studies are needed on a larger group of each coping style and should be replicated to confirm. Second, several studies have shown that the cortisol response is affected by a variety of factors, including age, gender, and other personal preferences.^22^, ^23^, ^24^ However, we did not consider gender differences. Additionally, we did not obtain other detailed private information, such as physical exercise, the timing of sleep, habitual intake of medicine, and body mass index. Third, we did not consider the possibility of coping flexibility. Students may adapt multiple coping styles and choose them according to the situation. As coping flexibility might be difficult to assess accurately, we did not assess it in this study. Finally, we did not conduct a cross‐cultural study, and there may be differences between Japanese (Eastern bias) and European people (Western bias). For example, Japanese people tend to have a lower sense of personal control and negative mood compared with British people.^25^, ^26^

## 5 CONCLUSIONS

In conclusion, the mean salivary cortisol concentrations of students with higher Likert‐type scores using the problem‐focused coping style were significantly lower than those of students with lower Likert‐type scores using the same coping style. The difference in mean cortisol concentrations between the two groups increased over time. Our findings suggest that salivary cortisol is a potential predictor for a problem‐focused coping strategy; monitoring students' salivary cortisol concentrations could identify stress‐related warning signs in this population.

## AUTHOR CONTRIBUTIONS

**Mitsuo Nagane**: Data curation; investigation; project administration; resources; writing—original draft. **Yoshinori Oyama**: Conceptualization; data curation; project administration; resources; supervision. **Fuminobu Tamalu**: Data curation; formal analysis; software. **Naofumi Miwa**: Conceptualization; formal analysis; methodology; project administration; supervision; writing—review and editing.

## CONFLICT OF INTEREST STATEMENT

The authors declare no conflict of interest.

## TRANSPARENCY STATEMENT

The lead author Mitsuo Nagane, Naofumi Miwa affirms that this manuscript is an honest, accurate, and transparent account of the study being reported; that no important aspects of the study have been omitted; and that any discrepancies from the study as planned (and, if relevant, registered) have been explained.

## Data Availability

The data that support the findings of this study are available from the corresponding author upon a reasonable request. Data derived from public domain resources.

## COPING STYLES QUESTIONNAIRE

### 1

1. The current situation will turn out all right.
2. Try to forget the whole thing.
3. Encourage myself to try my best.
4. Feel bad that I could not avoid the problem.
5. See the bright side of things.
6. The situation will improve with time.
7. Ask a friend to solve the problem.
8. Think it's not a big deal.
9. Find the cause of the problem.
10. Wait for something to be done.
11. Ask a friend about my situation.
12. Gather information about the problem.
13. Give up thinking that there are things like this.
14. My experience will help me.

Item number for

Problem‐focused coping style (5 items): 1, 7, 9, 11, 12.

Emotion‐focused coping style (3 items): 3, 5, 14.

Escape‐focused coping style (6 items): 2, 4, 6, 8, 10, 13.
